# Correction: Relative differential closure in hardy fields

**DOI:** 10.1007/s00605-026-02190-6

**Published:** 2026-07-23

**Authors:** Matthias Aschenbrenner, Lou van den Dries, Joris van der Hoeven

**Affiliations:** 1 Kurt Gödel Research Center for Mathematical Logic, Universität Wien, 1090 Wien, Austria; 2 Department of Mathematics, University of Illinois at Urbana-Champaign, Urbana, IL 61801 USA; 3 CNRS, LIX (UMR 7161), Campus de l’École Polytechnique, Palaiseau, 91120 France

**Correction to: Monatsh Math (2025) 208:541–568** **https://doi.org/10.1007/s00605-025-02077-y**

The proof of the important Lemma 4.10 in [3] used Lemma 1.7 from that paper, and the latter was taken from the online preprint [4] where it appears as Corollary 2.5.5. While preparing the monograph version [5] of that preprint we noticed a mistake in that corollary. Below we correct Lemma 1.7 and indicate how to fill the ensuing gap in the proof of Lemma 4.10.

**Correcting Lemma** 1.7

Let *K* be a differential field with algebraically closed constant field *C* and divisible group $K^\dagger$ of logarithmic derivatives. Let ${\operatorname {U}}$ be the universal exponential extension of *K*, with differential fraction field Ω. Suppose that *K* is $\operatorname {d}$-valued of *H*-type with $\Gamma \ne \{0\}$ and with small derivation. The correct version of [3, Lemma 1.7] now reads as follows:

## Lemma 1.7

Suppose *K* is λ-free and $\operatorname {I}(K)\subseteq K^\dagger$. Let Ω be equipped with a spectral extension of the valuation of *K*. Then the *H*-couple $(\Gamma _\Omega ,\psi _\Omega )$ of Ω is divisible and has asymptotic integration with $\Psi _\Omega :=\big \{ \psi _\Omega (\gamma ):\gamma \in \Gamma _\Omega ^{\ne }\big \}\subseteq \Gamma$.

(We drop the claim that the *H*-couple $(\Gamma _\Omega ,\psi _\Omega )$ is closed.) In the rest of this note we show that this weakened version of Lemma 1.7 suffices to prove Lemma 4.10. First we generalize [1, 9.9.2].

**Generalizing** [1, **9.9.2**]

Let ${{\boldsymbol{k}}}$ be an ordered field. We work in the setting of *H*-couples over ${{\boldsymbol{k}}}$ as in [2] and freely use terminology from that paper. (The application to Lemma 4.10 only needs the case ${{{\boldsymbol{k}}}= {\mathbb Q} }$.) Let $(\Gamma ,\psi )$ be an *H*-couple over ${{\boldsymbol{k}}}$. We extend $\psi :\Gamma ^{\ne }\rightarrow \Gamma$ to a function $\psi :\Gamma _\infty \rightarrow \Gamma _\infty$ by $\psi (0)=\psi (\infty ):=\infty$. For $\alpha _1,\dots ,\alpha _n\in \Gamma$, n⩾1, we define $\psi _{\alpha _1,\dots ,\alpha _n}:\Gamma _\infty \rightarrow \Gamma _\infty$ by recursion on *n*:

$$\psi _{\alpha _1}(\gamma ):= \psi (\gamma -\alpha _1),\quad \psi _{\alpha _1,\dots ,\alpha _n}(\gamma ):= \psi \big (\psi _{\alpha _1,\dots ,\alpha _{n-1}}(\gamma )-\alpha _n\big ){\text { for}}\, n\geqslant 2.$$

We now have the following strengthening of [2, Proposition 6.3]. (Note that [1, 9.9.2] is [2, Proposition 6.3] for the case ${{\boldsymbol{k}}}= {\mathbb Q}$.)

## Lemma 0.1

Suppose $(\Gamma , \psi )$ has asymptotic integration and let $(\Gamma ^*,\psi ^*)$ be an *H*-couple over ${{\boldsymbol{k}}}$ extending $(\Gamma ,\psi )$. Suppose n⩾1, $\alpha _1,\dots , \alpha _n\in \Gamma$, $c_1,\dots , c_n\in {{\boldsymbol{k}}}$ and $\gamma \in \Gamma ^*$ are such that

$$\begin{aligned} \psi ^*_{\alpha _1,\dots ,\alpha _n}(\gamma )&\ne \infty \quad ({\text {so}}\, \psi ^*_{\alpha _1,\dots ,\alpha _i}(\gamma )\ne \infty \, {\text {for}}\, i=1,\dots ,n),\ {\text { and}}\\ \gamma + c_1\psi ^*_{\alpha _1}(\gamma )&+ \cdots + c_n\psi ^*_{\alpha _1,\dots ,\alpha _n}(\gamma )\in \Gamma . \end{aligned}$$

Then $$\gamma \in \Gamma $$.

## Proof

Replacing $(\Gamma ^*,\psi ^*)$ by a closed *H*-couple over ${{\boldsymbol{k}}}$ extending $(\Gamma ^*,\psi ^*)$, we arrange $(\Gamma ^*,\psi ^*)$ to be closed. Then the *H*-triple $(\Gamma ^*,\psi ^*,\Psi ^*)$ over ${{\boldsymbol{k}}}$ extends $(\Gamma ,\psi ,\Psi ^\downarrow )$, and $(\Gamma ,\psi ,\Psi ^\downarrow )$ is definably closed in $(\Gamma ^*,\psi ^*,\Psi ^*)$ by [2, Proposition 6.1]. Now use that with $D^*_\alpha :=\big \{\gamma ^*\in \Gamma ^*:\psi ^*_{\alpha _1,\dots ,\alpha _n}(\gamma ^*)\ne \infty \big \}$, the function

$$\gamma ^*\mapsto \gamma ^* + c_1\psi ^*_{\alpha _1}(\gamma ^*) + \cdots + c_n\psi ^*_{\alpha _1,\dots ,\alpha _n}(\gamma ^*)\ :\ D^*_\alpha \rightarrow \Gamma ^*$$

is strictly increasing by [2, Lemma 6.2]. □

**Repairing the proof of Lemma 4.10**

Replace the end of the proof, starting with “By [1, 14.2.5] ...”, by the following:

By [1, 14.2.5] we have $\operatorname {I}(K)\subseteq K^\dagger$, so the *H*-couple of Ω is divisible and has asymptotic integration with $\Psi _\Omega \subseteq \Gamma$, by Lemma 1.7. Hence $\gamma \in \Gamma _\Omega$ by Lemma 0.1 above. Together with $\Psi _\Omega \subseteq \Gamma$ and $\alpha _{m+1},\dots ,\alpha _n\in \Gamma _\Omega$ this gives ${\psi _{\alpha _{m+1}}(\gamma ),\dots , \psi _{\alpha _{m+1},\dots ,\alpha _n}(\gamma )\in \Gamma }$ and thus $\gamma \in \Gamma$, a contradiction.

## Data Availability

This paper has no associated additional data.
